# *“I’m not strong enough; I’m not good enough. I can’t do this, I’m failing”-* A qualitative study of low-socioeconomic status smokers’ experiences with accesssing cessation support and the role for alternative technology-based support

**DOI:** 10.1186/s12939-017-0689-5

**Published:** 2017-11-13

**Authors:** Veronica C. Boland, Richard P. Mattick, Hayden McRobbie, Mohammad Siahpush, Ryan J. Courtney

**Affiliations:** 1 University of New South Wales (UNSW), National Drug and Alcohol Research Centre (NDARC), 22-32 King Street, Randwick, NSW 2031 Australia; 2 Wolfson Institute of Preventive Medicine, Queen Mary University of London, EC1M 6BQ, London, UK; 3 Department of Health Promotion, Social and Behavioral Health, University of Nebraska Medical Center, Omaha, NE USA

**Keywords:** Smoking cessation, Qualitative, Cessation support, Electronic cigarettes, mHealth

## Abstract

**Background:**

The social gradient in smoking rates persist with an overrepresentation of smoking and its associated harms concentrated within lower socioeconomic status (SES) populations. Low-SES smokers are motivated to quit but face multiple barriers when engaging a quit attempt. An understanding of the current treatment service model from the perspectives of treatment-seeking low-SES smokers is needed to inform the design of alternative smoking cessation support services tailored to the needs of low-SES populations. This qualitative study aimed to: i) explore low-SES smokers’ recent quitting experiences; ii) assess factors that impact treatment engagement; and iii) determine the acceptability and feasibility of alternative approaches to smoking cessation.

**Method:**

Low-SES participants (*n* = 24) previously enrolled in a smoking cessation RCT participated in either a semi-structured focus group or in-depth telephone interview. Data was obtained and analysed using thematic analysis from October 2015 to June 2016. Analysis was deductive from the interview guide and supplemented inductively.

**Results:**

Participants expressed feelings of guilt and shame around their smoking behaviour and experienced stigmatisation for their smoking. Guilt, shame, and stigmatisation negatively impacted treatment seeking behaviours with most avoiding current quit services. Costs of pharmacotherapy and treatment adherence were commonly cited barriers to treatment success. Electronic-cigarettes were perceived to be unsafe due to uncertainty on their legal status and regulatory restrictions. Technology-based text-messaging quit support was endorsed as a more favourable alternative compared to existing behavioural treatment services.

**Conclusion:**

Stigmatisation was commonly endorsed and acted as an impediment to current treatment utilisation. Electronic-cigarettes may present a viable harm reduction alternative, but their likely uptake in socioeconomically disadvantaged groups in Australia is limited by smokers’ uncertainty about their regulation and legality. Mobile phone based cessation support may provide an alternative to telephone counselling and overcome the stigmatisation low-SES smokers face while trying to quit.

## Background

Smoking prevalence in Australia (i.e. daily smokers aged 14 years and older) is currently below 13% and has declined from 24.3% in 1991 [1]. The reduction in the Australian smoking rate has largely emanated from Government policy [2], in particular strong enforcement of measures outlined in the World Health Organization’s (WHO) Framework Convention on Tobacco Control (FCTC) [3]. Despite this decrease, smoking rates remain disproportionately high among smokers from low socioeconomic status (SES) backgrounds; 19.9% in the lowest-SES bracket compared to 6.7% in the highest-SES bracket [1]. Low-SES can be characterised by low educational attainment, low incomes, unemployment and include the long-term unemployed, homeless, mentally ill, ethnic minorities, prisoners, at-risk-youth and single parents [4].

While Australian tobacco control policies such as increasing the cost of tobacco and banning smoking in public areas has led to an overall decline in smoking rates, such policies may have had unintended consequences among disadvantaged groups who continue to smoke at high rates. Low-SES smokers often have higher levels of nicotine dependence [5] are just as motivated and try to quit as their high-SES counterparts but are less likely to succeed during a quit attempt [6]. Factors that may contribute to this reduced success is the social context of smoking where smoking behaviours are entrenched and normalised [7, 8] and stigmatisation of this health risk behaviour [9]. Smoker-related stigma encourages secrecy and social withdrawal from non-smokers [10] and further exacerbates health inequalities since smoking prevalence is highest amongst socioeconomically disadvantaged smokers [11, 12]. Among other population groups with health risk behaviours or chronic diseases including persons with a mental health disorder, hepatitis C, HIV, sex workers, and injecting drug users’ stigmatisation functions as a barrier to help-seeking behaviours [13–17]. However, the subjective experience of smoker-related stigma among low-SES smokers is not well understood and needs further attention.

A factor contributing to the high smoking rates among disadvantaged groups is the overall scarcity of research output targeting disadvantaged smokers [18] and the widespread paucity of evidence for effective smoking cessation interventions for disadvantaged smokers [19]. Engaging with low-SES smokers to discuss alternative cessation support is needed if smoking rates are to decline for this group. Providing tailored support to meet the needs of low-SES is urgently needed and requires consumer engagement at all stages of intervention development.

Potential alternatives include the use of electronic cigarettes (ECs) and technology-based behavioural support. A recent Cochrane review found ECs to be an effective cessation device compared to placebo, and that ECs were effective at reducing cigarette consumption in smokers unable to quit [20], an approach that may be underutilised. Since mobile phone technology penetration and accessibility is high among disadvantaged groups [21] behavioural support delivered via mobile phones could be a viable alternative. The *WHO Tobacco Free Initiative* identified mHealth as a cost-effective, scalable, and sustainable platform for tobacco control interventions [22], however, it is not known qualitatively if cessation support delivered via mobile-phone technology is acceptable among disadvantaged smokers.

Although engaging low-SES smokers after they have attempted to quit smoking provides an opportune time to explore the personal experiences of a quit attempt in order to gain feedback for future research, this approach is lacking. Gaining feedback on alternative quit support may serve to benefit future intervention designs aimed at increasing cessation among low-SES smokers. This qualitative study served to fill this research gap and aimed to: i) explore low-SES smokers’ recent quitting experiences; ii) assess the factors that impacted treatment engagement and quit success immediately following participation in a large-scale randomised controlled trial (RCT) [23]; and iii) determine the acceptability and feasibility of alternative approaches to cessation including electronic cigarettes and technology-based support.

## Method

### Design

Participants who had previously participated in a smoking cessation RCT aimed at improving smoking cessation outcomes in low-SES smokers by providing financial education and support to reduce financial stress [23] between 2013 to 2015, were invited to participate in this qualitative study. Further details on the RCT are available elsewhere [23]. A combination of focus groups (FGs) and in-depth interviews were used to provide a comprehensive exploration of the complexities of quitting faced by this disadvantaged group. FGs allow for participant interactions as a way to stimulate discussions and gain multiple perspectives and the sharing of ideas and experiences that may not be explored in individual interviews [24] while individual telephone interviews sought to enhance data richness by drawing on personal in-depth accounts that might not be disclosed in a group setting [25].

Three focus groups (FGs) were conducted at the University of New South Wales (UNSW) campus (*n* = 2) or at a community library (*n* = 1), and seven in-depth interviews were conducted over the telephone in UNSW interview rooms in Metropolitan Sydney, Australia. All participants were reimbursed $50 for their time. The study received ethics approval from the UNSW Human Research Ethics Committee (HC15523), and was funded by the Cancer Institute New South Wales (CINSW), Australia.

### Participant eligibility

Participants who had participated in a previous RCT [23] and consented to being contacted for future research were eligible to participate in this study. Inclusion criteria for the previous RCT was: aged 18 years or over; currently smoking at least 10 cigarettes per day; currently in receipt of a government pension or allowance (proxy for low-SES); motivated to quit; willing to make a quit attempt in the next month; not currently taking any smoking cessation medications; willing to receive telephone-based support to help quit smoking; able to read and understand English language; and have a home or mobile telephone. All participants in the previous RCT were mailed an 8-week supply of combination NRT comprising 21 mg/24-h nicotine patches plus either 2 mg gum or lozenges.

Due to geographical constraints and travel requirements only participants residing within the Sydney region were invited to participate in FGs. Sydney region was defined by suburb and postcode based on the Australian Government’s Department of Social Services statistical division postcode reference list (https://www.dss.gov.au/our-responsibilities/settlement-and-multicultural-affairs/programs-policy/settlement-services/settlement-grants-program/assistance-from-the-department/publications/statistical-divisionpostcode-reference-list). In-depth interviews were restricted to participants who had completed final follow-up in the previous RCT within a year of this qualitative interview being conducted.

### Focus groups

A total of 133 prospective participants resided within the Sydney region and were sent an invitation letter. Of the 133 participants, 64 (48%) were unable to be contacted, 35 (26%) were not interested in participating, and 12 (9%) were unable to travel to the specified locations. A total of 22 accepted the invitation to participate, but five did not attend leaving a total of 17 participants. Reasons were not provided for non-attendance. All FG participants provided written informed consent and each FG was audio recorded. FGs included between four to eight participants, and ran for 90 min.

### In-depth telephone interviews

Individual interviews were conducted after the FGs were completed. Invitation telephone calls were conducted to outline the study and participation requirements. If the invitation was accepted, information sheets were mailed to participants, and verbal consent was obtained at the beginning of the interview. A total of 12 invitation telephone calls were made, of which five were non-contactable, and seven were accepted. All 60-min in-depth interviews (*n* = 7) were audio-recorded. Four of the seven participants were biochemically verified abstinent but at the time of the interview one participant had relapsed.

### Procedure

FGs and in-depth interviews were conducted in 2015 and facilitated by two members (VCB and RJC) of the research team, one researcher (VCB) was trained in qualitative research methods. Both researchers were involved in the previous RCT. A semi-structured interview guide was used to facilitate discussions and ensure the same topics were covered in FGs and in-depth interviews but were not followed prescriptively. Questions were designed to explore participants quitting experiences, drawing on their engagement with quit support treatment and services, the factors that influence their treatment engagement, and to open discussion about the type of support they want and recommendations they have for a future research project.

### Measures

Socio-demographic, smoking characteristics, and quitting behaviours including age, sex, marital status, education, number of cigarettes smoked pre and post RCT, number of years smoked and percent of life smoked, ever tried to quit and use pharmacotherapy, and ever spoken to the Quitline was obtained from the data collection in the previous RCT [23]. The previous RCT used the Russell Standard criteria [26] using cotinine urine analysis to verify prolonged abstinence. Current smoking status was obtained via self-report (i.e. *Are you currently smoking?* yes/no). Participants were also asked if they owned a mobile phone and used the text-messaging function at least once a week.

### Data analysis

Audio-recordings were transcribed verbatim and checked against audio recordings to ensure accuracy and overview of the text. FG and in-depth interviews were analysed together using Braun and Clarke’s six stage method for thematic analysis [27]. VCB read all transcripts several times to familiarize with the content. Analysis was deductive from the interview guide and supplemented inductively. Initial codes were generated based on interview guide headings and patterns observed in the data with first order concepts or codes including “strategies/approaches to quitting” identified. Smoker and ex-smoker experiences were grouped separately by these codes. Codes were then grouped into overarching themes, and sub-themes were identified within these themes. Some sub-themes also had key concepts. Formatted transcripts were imported into the computer software NVivo 10 (QSR International, Melbourne, Australia). Thematic maps were generated to assist in understanding relationships of themes [28, 29] and were developed using the scissor-and-sort technique [30]. Data analysis was performed by VCB who met regularly with RJC throughout the coding process to discuss and refine themes and sub-themes. VCB and RJC were researchers on the previous RCT which provided contextual information while analysing the data. Participant quotations were chosen to illustrate themes, sub-themes and key concepts with participant identifiers being sex (F: female or M: male), and smoking status (smoker or ex-smoker). Descriptive analysis of demographic data was performed using statistical software package STATA, version 11.

## Results

### Sample

Table 1 reports the participant characteristics by smoking status and total sample. A total of 24 participants, 12 males and 12 females, took part in either a mixed gender FG or in-depth interview. The average age of the total sample was 48 years (SD = 14.1), 45.8% had completed high school or less, and 87.5% were single and lived alone. At the time of interviewing, a total of five (20.8%) participants self-reported quit status and 22 (92%) participants owned a mobile phone with 19 (79.2%) participants sending text-messages at least once a week.

**Table 1:** Sample characteristics (*n* = 24)

	Smoker (*n* = 19)	Ex-smoker (*n* = 5)	Total (*n* = 24)
Gender (n, %)			
Male	11 (57.9)	1 (20)	12 (50)
Female	8 (42.1)	4 (80)	12 (50)
Age(M,SD)	57.7 (13.4)	34.4 (6.8)	48.1 (14.1)
Married/living with a partner (n,%)	1 (5.1)	2 (40)	3(12.5)
Single/sole parent/divorced/live alone (n,%)	18 (94.7)	3 (60)	21 (87.5)
Education (high school or less) (n,%)	8 (42)	3 (60)	11 (45.8)
Cigarettes smoked per day before entering RCT^a^ (M,SD)	20.9 (9.4)	27 (15.5)	22.2 (10.9)
Cigarettes smoked per day at end of RCT^ab^ (M,SD)	8.1 (9.0)	0	6.6 (8.7)
Russell Standard abstinence at end of RCT^a^ (n,%)	0	4 (57.1)	4 (16.7)
Self-reported abstinence at time of FG/interview (n,%)	0	5 (100)	5 (20.8)
No. of years smoked^a^ (M,SD)	34.6 (14.5)	18.6 (8.9)	31.3 (14.9)
% years of life smoked^a^ (M,SD)	64.5 (12.5)	51.1 (18.9)	61.7 (14.7)
Ever tried to quit smoking before RCT^a^ (n, %)	17 (89.5)	5 (100)	22 (91.7)
Ever tried NRT to quit before RCT^ab^ (n, %)	12 (70.6)	4 (80)	16 (72.7)
Ever tried prescription medication to quit before RCT^ab^ (n, %)	5 (26.3)	1 (20)	6 (25)
Spoken to Quitline end of RCT^ab^ (n, %)	16 (84.2)	3 (60)	19 (82.6)
Owned a mobile phone (n,%)	17 (89.5)	5 (100)	22 (91.7)
Sent texts at least once a week (n,%)	14 (73.7)	5 (100)	19 (79.2)

^a^Data obtained from RCT database

^b^not all participants completed this question (*n* = 22)

### Themes, sub-themes, and key concepts

Two broad themes (treatment acceptance barriers and alternative cessation support), four sub-themes, and 13 key concepts were identified. A summary of the themes, sub-themes, key concepts, and sample quotes are shown in Table 2.

**Table 2:** Index of themes, sub-themes, and key concepts

Theme	Sub-theme	Key concept	Exemplar quotation
Treatment acceptance barriers	Stigmatisation	Shame	“And shame - I mean, it is so unacceptable, and I just have to be very secretive about it. I would never let anybody I work with know that I smoke…it’s just so shameful.”(F, smoker)
		Tobacco control policies	*“So although the actual bans now- there are so many places that you cannot smoke…it also makes you zero in on that freedom to get to your own house and go into your own backyard and have a little cigarette. And that’s like some part of control that you’re taking back.” (F, smoker)*
	Pharmacotherapy treatments	Cost	*“If you don’t believe that it’s going to work and you think, well next week I’ll just go back to buying cigarettes again, then it is more expensive.” (F, ex-smoker)*
		Subsidised medication	“I’m just waiting for my time to come up. I’ve just been to the doctor now and he says, ‘No, no, you’re still got three months to go (before PBS entitlement is renewed) .”
		Adherence	“I think for the first two weeks of taking it [varenicline], I had a couple of cigarettes, then after that nothing. But my unfortunate thing is the three times I have had it, I haven’t gone the whole way with the tablets…I start smoking again” (F, smoker)
		Withdrawal	*“If I'm trying to get off a dependency on cigarettes, well I don’t want to be dependent on a (nicotine) gum, I don’t want to be dependent on Champix medication or whatever it is, or e-cigarettes or any kind of replacement.” (F, smoker)*
		Side-effects	*“I started getting an itchy red spot or rash from the patches. So I went back to smoking.” (F, smoker)*
Alternative cessation support	Electronic cigarettes	Knowledge	“I find it hard to get any real information about them. Even if you go into a tobacconist that sells them and you say, ‘Well, tell me about them,’ they don’t really know.” (M, smoker)
		Purpose/function	*“It’s still smoking. No. What’s the difference between lighting a smoke or inhaling on an e-smoke?” (F, ex-smoker)*
		Concerns	*“They say it could take up to 30 years to know what the effects of the additives they put in for the flavouring. I mean, it’s like well, am I swapping one bad and then adding another?” (M, smoker)*
	Mobile phone behavioural support	Effectiveness	*“Yeah. I think it’s [text support] probably a little bit better than a phone call because it’s a little bit more personal….everyone uses their phones these days, so yeah… it’s a personal conversation.” (F, ex-smoker)*
		Tailored interactive support	“I think it’s important to emphasise that it would be interactive texting as opposed to just receiving a message.” (F, smoker)
		Content	“The health benefits I’d like just to come up after a specific time. And money saved. And you’ve also saved this much. I would be happy to know that.” (M, smoker)

## Treatment acceptance barriers

### Stigmatisation

#### Shame

Participants expressed feelings of guilt and shame around their smoking behaviours and society’s perceptions of them as smokers. The disapproval they have of their own smoking behaviours combined with the perceived disapproval they experience when accessing treatment was expressed by the shame they felt when having to admit their smoking addiction to someone else: “*Exactly, exactly. Because* [if Quitline calls] *they’re like, oh I'm smoking. This is just shameful*.” (F, ex-smoker).

Shame was linked to previous failed quit attempts, the belief that they have failed to control their addiction and were a slave to nicotine, failure at needing external support to combat this addiction, and the futility of trying to quit when they considered smoking as entrenched in their social networks. Also, failing to quit while using pharmacotherapies was also expressed and most cited that nicotine was harder to quit than heroin and therefore quitting smoking was perceived as impossible: “*It’s a joke, because it’s like treating a drug dependency for heroin with small doses of heroin. That’s exactly what it is. All the smokers that tried to quit that I know, about 15 people, all of them tried patches and chewing gum. None of them quit.”* (M, smoker).

Factors that reinforced the shame and stigma they experienced included past interactions with Quitline telephone counselling. Quitline was perceived to be counter-productive and condescending. Not calling Quitline or telling friends and family they were trying to quit was a shame-saving strategy: “*Because if you’re calling Quitline then you’re accepting defeat of some form. People just don’t like doing that stuff*.” (M, ex-smoker); “*I never tell people that I have quit smoking. I have at times… the longest I’ve gone without smoking would be about two years and during that two years people say, ‘I heard you’ve quit smoking,’ and I’d say, ‘No, no. I'm just not smoking at the moment,’ because I cannot bear the thing, that shame of them then seeing me again smoking, you know what I mean. Oh I failed again*!” (F, smoker). The main reasons for not liking Quitline were: they felt like they were being monitored or judged; they felt the service reinforced the pressure to succeed and increased the chances of feeling shame if they failed or guilty if they slipped up; and they simply did not like the strategies and feedback they received from Quitline counsellors: *“The first time I rang Quitline the young lady told me to have a carrot!... And that’s why I dropped off and I went back to smoking.”* (M, smoker); “*I just didn’t like it* [Quitline] *because it made me feel guilty…..It made me feel guilty, so I hated it*.” (F, ex-smoker).

#### Tobacco control policies

Tobacco control policies and reforms such as banning smoking in public spaces, increased tobacco taxes, and the societal changes in attitudes towards smoking directly impacted participant’s everyday lives and quit attempts. Issues discussed focussed on: personal experiences of being vilified or abused in public: “*I was walking around near where I live and there is an outside dining area, and I was walking along with a cigarette talking on my phone and someone at the table screamed out, ‘You can’t smoke four metres from food being served*.’” (M, smoker); the internalisation of the stigma associated with being a smoker: “*Even when I started smoking – that was 20 years ago – it was… there were more provisions for smokers than there were non-smokers; there were never any non-smoking areas*…[the societal change in how we treat smokers]… *That’s where the stigma comes from*.” (M, smoker); and the negative impact of increased tobacco taxes preventing spontaneous quit attempts: “*The economics of smoking. If you buy a pack and you have a quit date set up, if you’ve still got three or four left at the end of the pack you don’t want to throw them out [equivalent to throwing money away].”* (M, smoker).

### Pharmacotherapy treatment

#### Cost

The cost associated with buying cigarettes and over-the-counter (OTC) NRT was considered to be comparable. The lack of confidence expressed in using pharmacotherapy treatment meant that participants would rather spend their money on cigarettes, which they enjoyed, than pay full price for a medication that may or may not work. Therefore, the cost of purchasing NRT was considered in the event they relapsed prohibitively expensive due to the combined cost of NRT and a packet of cigarettes: *“You’re buying the same amount [of NRT] as a packet of cigarettes so what’s the use if you’re not smoking [you’re still spending the same amount].”* (F, smoker).

#### Subsidised medication

Accessing Australian Government subsidised smoking cessation medication through the pharmaceutical benefits scheme (PBS) is restricted to one course of treatment every 12-months. Due to the entitlement restrictions, some participants expressed planning their quit attempts around their yearly PBS prescription and did not consider unplanned or unassisted quitting as a feasible option for them: *“The cost of different products. Like, not everyone can afford to, like, either have it privately or under PBS, not everyone can do it on PBS. And the cost of it, sometimes it costs more to quit than it does to actually smoke*.” (F, smoker).

#### Adherence

Non-adherence to medications presented as a barrier to cessation and was viewed as a contributing factor for relapse. Most participants indicated they had ceased treatment early because they forgot to take their medication, stopped due to side-effects, or they believed they did not need it anymore either because of relapse or cessation: *“Yeah, I just forgot to take it [varenicline]. Because I'm not really big on taking tablets, so my memory for taking tablets – it just goes out the door*.” (F, ex-smoker). Often participants did not use smoking cessation medication at the recommended dose, despite acknowledging that the treatment was effective when used correctly: “*It would do if I used it correctly. I mean, I do use the patches and I still smoke while I’ve got patches on* **.”** (F, smoker).

#### Withdrawal

A common belief was that NRT was delaying the inevitable nicotine withdrawal and was creating another addiction to overcome. Participants expressed cessation as eliminating all nicotine. For some participants NRT presented a barrier to quit success since it still meant they were dependent on nicotine: *“I found that I'm just postponing the inevitable. The nicotine withdrawal’s going to come as soon as you take that last patch off, well it was for me. Even weaning down a bit, when I jumped off that last bit it felt the same as just putting cigarettes down*.” (M, ex-smoker).

#### Side-effects

Most participants cited side-effects as the cause for non-adherence to medications (i.e. NRT or varenicline). Side-effects ranged in severity from skin rashes to psychological disturbance: *“Like after four days of taking it [vareniclicne] I had to call my GP and say, ‘Is this supposed to happen?’ And he said, ‘Ok, immediately stop using it. Come in and see me.’”* (M, smoker). Theyalso cited smoking concurrently while using NRT or varenicline. It appeared that pharmacotherapies were effective at reducing cigarette consumption but not complete cessation for some: *“While I was on the nicotine patches I used to also smoke at the same time.”* (M, smoker).

## Alternative cessation support

### Electronic cigarettes (ECs)

#### Knowledge

Most participants knew of ECs but wanted more information about them: *“I have heard of them. That’s the electronic one that actually feels like you’re smoking but it’s got nothing in it or something*.” (F, smoker).

#### Purpose/function

Some participants had tried ECs but it was not clear if the device they had used contained nicotine or not. There were differing views regarding the purpose and use of ECs with some participants viewing them as equivalent to smoking or as a potential cessation aid like NRT: *“I was like, wow, this is stupid. This is more work for people that want to give up*.” (F, ex-smoker)*.*

#### Concerns

The main concerns expressed with using ECs centred on safety and efficacy and whether such devices were legal in Australia. Although some viewed ECs as a potential cessation aid, overall ECs were perceived as comparable to smoking cigarettes with participants not inclined to use or recommend their use: “*Are they legal? If it is illegal and I had to do it on the Internet I wouldn’t do it. That’s another thing – cigarettes are still a legal substance. Yep, so if it was illegal I probably wouldn’t do it*.” (F, smoker).

### Mobile phone behavioural support

#### Effectiveness

Participants expressed the need for alternative support services and were receptive to technology-based quit support in the form of mobile phone text-messaging with very few suggesting smart-phone applications: *“If I was getting a text message when I was feeling vulnerable, it could probably turn me a way [from smoking]*.” (F, smoker).

#### Tailored interactive support

Most participants expressed the need for interactive tailored support and suggested intensive text-message support within the first two weeks of quitting: “*I think someone has to be there to message you back at that time, whatever time it is. Twenty-four hours.*...” (F, smoker). Text-message quit support was viewed more positively than traditional telephone counselling. This was largely due to the perception that they would have more control over their support and could decide when and where they engaged with text-quit support. Text-messaging also provided the potential for 24/7 ‘real-time’ support and could remain hidden from friends and family: “*I mean, you don’t have to tell anyone who you’re texting or whatever*.” (F, ex-smoker).

#### Content

The content of text-messages was focussed on the provision of ‘suggestions’, positive expectancies, and reminders to take medication: “*Knowing the progress you’ve made as opposed to being constantly reminded that you’re a smoker.*” (M, smoker). Unhelpful texts were seen to focus on the negatives of smoking, either the harms associated with smoking, or the money lost from smoking. Participants expressed that they already knew the negatives of smoking and that it was counter-productive to be reminded of all the negative side-effects associated with smoking. The aim of text-support was to provide support in a way to allow them to feel like they were quitting on their own and that they weren’t failing themselves e.g. frame support as a ‘suggestion’ so they can decide how and whether they take that information on board: *“I don’t like people telling me what to do. But if they were to in a way where it doesn’t sound like they’re telling me what to do, even though they actually are, that works. ‘This is just a suggestion. It’s all suggestion; you don’t have to do it.”* (M, ex-smoker).

## Discussion

### Summary of main findings

This study engaged a unique sample of treatment seeking low-SES smokers who had recently embarked on a quit attempt, and obtained their subjective experiences of this attempt. All of the low-SES smokers and ex-smokers participating in this study had previously tried to quit and several factors preventing engagement with current behavioural and pharmacological cessation treatments were identified. Smoking cessation treatments received mixed reactions with most participants reporting undesirable or unhelpful treatment experiences. Overall, participants were receptive and positive about the potential role of technology-based cessation support in the form of text-messages and provided feedback about its acceptability and possible content. There were mixed views on the use of ECs as a potential cessation aid with concerns raised over their safety and efficacy and the legality of their use within Australia. Overall, current treatment approaches were perceived as not well-suited to meet the needs of low-SES smokers. Nonetheless, on a positive note, low-SES smokers and ex-smokers provided insights on alternative treatment strategies, and outlined how these treatment approaches can assist in overcoming some of the barriers affecting treatment.

### Main themes

Prior research has investigated the effects of tobacco control policies on smoking prevalence and tobacco consumption [31–33]. However, the current study provides insight into how smokers subjective experiences of these policies affect treatment seeking behaviours. Our findings suggest that there is a complex interplay between the changes in tobacco control policies and reform and how participants perceive their smoking behaviour within the wider social context. The dominant anti-smoking public health discourse in Australia [2] shapes policies and treatment programmes, and people’s experiences with these policies and programmes shape their identity [34]. The internalised view that non-smoking is the norm conceptually positions ‘smoking and smokers’ as a negatively constructed group. Further to this, recent tobacco excise increases in Australia has promoted smoking as unaffordable which may further marginalise low-SES smokers [35]. Consequently, the stigmatisation of being a smoker and the associated feelings of shame, guilt, and failure shaped a relatively negative viewpoint on quitting, and acted as a key barrier to accept or engage with quit support and therefore prevented treatment seeking behaviours. Our findings provide insights that may help to serve policy makers both nationally and internationally when developing and implementing new tobacco control policies.

The experience of stigmatisation highlights the connection between treatment services and the experience of disenfranchisement. Rather than Quitline being viewed as a support service, treatment seeking to this service was likened to admitting failure. Accepting help functioned as a barrier because it reinforced the stigma associated with being a smoker. Participants expressed a desire to be left alone during a quit attempt which could be a reaction to tobacco control policies and societal judgements placed on low-SES smokers. This finding provides insight into why some low-SES smokers are reluctant to call telephone counselling hotlines and do not perceive such services as helpful. This finding may indicate that the current treatment service model could be reoriented to address the feelings of guilt, shame, and the stigmatisation associated with smoking to encourage wider reach and participation. Treatment engagement is disproportionately low among disadvantaged groups [36, 37] and these study findings may help to re-shape current service models to meet the needs of low-SES smokers.

Increasing quit rates is a key challenge for the wider tobacco control field. To date, smoking cessation randomised controlled trials (RCT) targeting smokers from low-SES populations have found no intervention effect on cessation outcomes [38–40]. While low-SES smokers are willing and motivated to try to quit [41] the probability of success is low [6]. Despite the majority of participants in this study being treatment failures, they were still motivated to quit but expressed the need for alternative cessation support options. Technology-based quit support was seen as a potential alternative that could compliment current treatment approaches. Text-messaging has the potential to be easily accessible and less judgemental and is supported by the *WHO Tobacco Free Initiative* [22].

Overall, the function of text-messaging was to provide intensive quit support within the first two-weeks of quitting. Adherence support via text-messaging could also be considered since most participants did not adhere to NRT beyond two-weeks. The importance of personalised and interactive text-messaging was seen as providing your own one-on-one quit buddy which could overcome the stigma and resistance to accept quit support. This study provides preliminary feasibility and acceptability findings of text-messaging quit support for low-SES smokers, which may be applicable to low-SES smokers in similar countries such as the United States and the United Kingdom. Developing interventions within a community-based participatory research framework [42] is relevant to researchers both nationally and internationally and our findings add to the knowledge base for researchers and policy makers.

Participants expressed a desire for text message content that demonstrated progress e.g. number of days quit or money saved, reinforcement of health benefits from quitting, and increased willpower, and medication reminders. Despite low-SES smokers and ex-smokers expressing a willingness and positive attitude towards the use of text-message support, there is a lack of methodologically rigorous text-message intervention studies aimed at increasing quit rates among disadvantaged groups [43, 44]. A recent systematic review and meta-analysis examining technology based smoking cessation interventions for disadvantaged smokers found that technology based quit support increased the odds of cessation at 18-months follow-up [44]. However, of the 13 included studies, only one study used mobile phone text messaging. Based on this systematic review and the current study’s findings, there is a need for future research to develop mobile phone based cessation support for low-SES smokers which may overcome social inequalities, increase treatment engagement and adherence. Further research on the effectiveness of text-message cessation support in combination with or without Quitline support is required for this group.

The proliferation and growing popularity of ECs as a harm-reduction or cessation aid in the US, UK, and New Zealand [45–47] was not reflected among smokers and ex-smokers in this study. This may be an artefact of Australia’s stringent tobacco control policy on ECs, where selling and the distribution of liquid nicotine is illegal [48]. When faced with purchasing cigarettes or an EC product, participants did not see the advantage in purchasing an illegal device, over legally available tobacco products. The illegality surrounding ECs may foster suspicion around their safety and efficacy and blurs their function as a potential cessation aid. Although Australia’s strong regulatory framework prohibits the sale of EC containing liquid nicotine, ECs may provide an alternative and less harmful nicotine delivery method [20]. However, while Australian Government policy bans the sale of liquid nicotine vaporising devices, the effectiveness of ECs as a harm reduction or cessation aid among low-SES Australians may not be known.

In contrast to ECs, pharmacotherapy treatments were seen as an essential component to a quit attempt. However, concerns about the cost of treatment and PBS restrictions were factors impacting quit attempts. PBS restrictions delayed participant’s future quit attempts to coincide with their yearly prescription entitlement renewal. Although prior research has identified the cost of NRT as a barrier to treatment [19], participants considered the cost of NRT and cigarettes to be comparable. However, NRT was unaffordable when they considered the combined cost of cigarettes and NRT in the event they lapsed or relapsed.

Lower rates of cessation among disadvantaged groups have been linked to poor treatment adherence [4, 49, 50]. Barriers to pharmacotherapy adherence included side-effects, safety concerns, and transferring one addiction for another, and these findings are supported elsewhere [19, 51]. Although most participants had heard of varenicline only a few had used this product with most expressing a willingness to try it in the future. Overall, pharmacotherapies were viewed positively and participants expressed a willingness to use these products in future quit attempts.

### Limitations

This study drew on the experiences of low-SES smokers and ex-smokers who had previously taken part in a smoking cessation trial and most were treatment failures. The method of recruitment limited our sample to treatment seeking individuals who resided in inner-city areas and were low-SES. Smokers from high-SES background were not included in this study and future research should consider whether the key findings from this study are shared by or differ from high-SES smokers. Such studies may be useful to guide the design and potential tailoring of cessation support services for smokers from all SES backgrounds. The perspectives expressed in this study may differ from opinions of smokers or ex-smokers who do not engage with treatment services and have a preference for unassisted methods. The views expressed may also differ among rural and remote smokers and ex-smokers and among other population groups where high smoking rates persist e.g. Indigenous populations, people experiencing homelessness, prisoner populations, and people living with HIV or severe mental illness. Interview methodology is a further limitation of the study. While face-to-face interviews can provide rich sources of data they are costly and can be a source of response bias [52], however, conducting telephone interviews can also lead to some information being lost due to the inability of the researcher to use visual help [53]. Future research may wish to analyse the data by interview type to see if patterns in the data differ by focus group and in-depth interview. Since participants were recruited from the previous RCT the researchers were involved in, participants were informed that their feedback will help guide a future quit project to overcome a desirability effect or social acceptability in reporting. Although every effort was made by the researchers to engage participants in a safe and non-judgemental environment, there is a possibility that some participants may have felt judged and modified their views to overcome this.

## Conclusions

This study drew on the day-to-day experiences of low-SES smokers and ex-smokers. It is evident that although tobacco control policies are effective at reducing overall smoking rates in Australia, such policies may present unintended consequences including stigmatisation, and further marginalisation of low-SES smokers. The provision of alternative harm reduction strategies is needed to reduce the disproportionately high rate of smoking in low-SES groups compared to their peers. Technology-based cessation support requires further investigation since it has the potential to meet the complex needs of low-SES groups by delivering cost-effective tailored support.
